# Capsular Outcomes Differ with Capsulorhexis Sizes after Pediatric Cataract Surgery: A Randomized Controlled Trial

**DOI:** 10.1038/srep16227

**Published:** 2015-11-05

**Authors:** Haotian Lin, Xuhua Tan, Zhuoling Lin, Jingjing Chen, Lixia Luo, Xiaohang Wu, Erping Long, Weirong Chen, Yizhi Liu

**Affiliations:** 1 The State Key Laboratory of Ophthalmology, Zhongshan Ophthalmic Center, Sun Yat-sen University, Guangzhou, Guangdong, 510060, People´s Republic of China

## Abstract

Capsular outcomes of anterior/posterior capsulorhexis opening (ACO/PCO) are essential for performing a secondary in-the-bag intraocular lens implantation. To compare the capsular outcomes with different primary capsulorhexis sizes, Thirty-eight eligible patients (45 eyes) were randomly assigned to three groups by anterior capsulorhexis diameter (Group A: 3.0–3.9, Group B: 4.0–5.0, and Group C: 5.1–6.0 mm). The areas of ACO/PCO and posterior capsule opening opacity (PCOO) as primary outcomes, while, the incidence of visual axis opacity (VAO) as secondary outcome were measured at follow-up visits. Among the thirty eyes included in the final analysis, the mean area of the ACO decreased significantly, whereas the PCO enlarged with time. Group A had the highest anterior capsule constriction and percentage reduction, which increased with time. There were significant differences in the percentage reductions at 6 months and 1 year compared to 1 month in Group A and B. Group C had the highest posterior capsule enlargement. The percentage of PCOO to PCO area and the incidence of VAO was highest in Group A and lowest in Group C. Thus, Capsulorhexis diameter of 4.0–5.0 mm may yield better capsular outcomes, considering moderate contraction of ACO, moderate enlargement of PCO, and lower percentage of PCOO and VAO.

Pediatric cataract is one of the common cause of childhood blindness and are responsible for approximately 10–20% of blindness in children worldwide^1^. Despite the rapid developments in recent years of techniques for pediatric cataract surgery^2^,^3^, many children experience limited vision improvement after intervention, due to various capsular complications. Visual axis opacity (VAO), anterior and posterior capsule fibrosis and capsule contraction are common capsular complications that result from the high proliferative capacity of the lens epithelium and severe postoperative inflammation. To reduce the high incidence of capsular complications and refractive uncertainties of early intraocular lens (IOL) placement techniques, many surgeons choose to leave infantile cataract patients aphakic before mature and stable development of the eye ball^4^,^5^,^6^.

It is well known that the pediatric eye is more reactive after cataract surgery^7^. An increasing number of pediatric ophthalmologists have recognized that the ideal site for IOL implantation is in the capsular bag, because in-the-bag implantation sequesters the IOL from the highly reactive uveal tissue and maintains better IOL centration^8^,^9^,^10^. In our previous study, we found that, prior to performing a secondary in-the-bag IOL implantation, an ideal capsular outcome for the anterior/posterior capsulorhexis opening (ACO/PCO) should be achieved after the primary surgery^11^. Although many studies of the impact of capsulorhexis diameter, location and shape on the development of posterior capsule opacification after age-related cataract surgeries have been conducted^12^,^13^,^14^, there are no reports investigating the relationship between primary capsulorhexis size and capsular outcome after pediatric cataract surgery. Therefore, the aim of the current study was to prospectively evaluate the capsular outcomes of three controlled groups of patients with different anterior capsulorhexis diameters (3.0–3.9, 4.0–5.0, and 5.1–6.0 mm).

## Results

**Figure 1:**
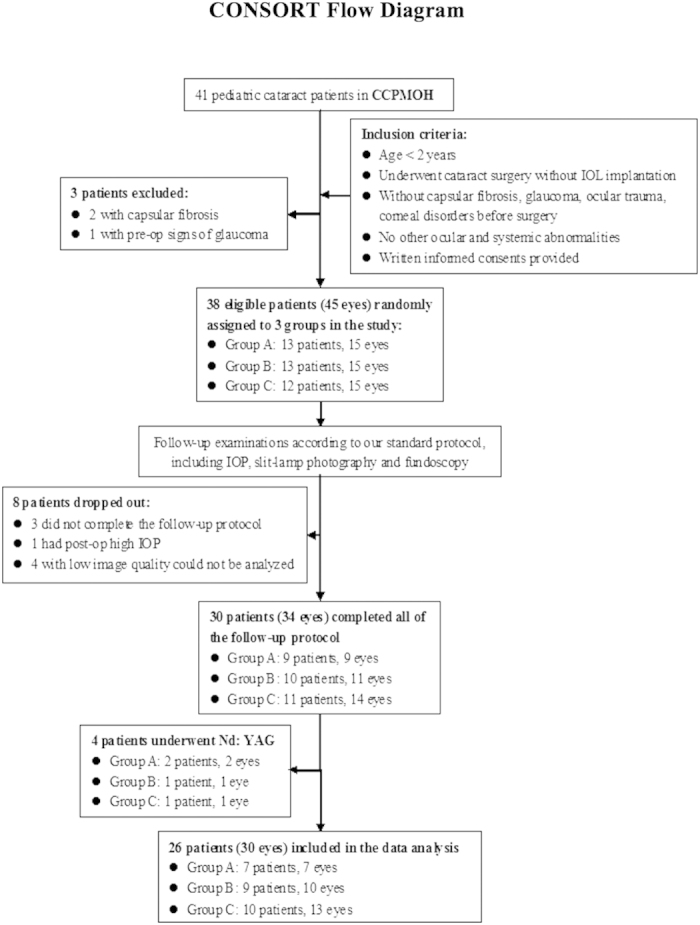
Flow chart of the patient selection and follow-up protocols. (Notes: CCPMOH = Childhood Cataract Program of the Chinese Ministry of Health; IOL = Intraocular lens; Nd: YAG = neodymium: Yttrium–aluminum–garnet laser capsulotomy).

In total, 8 patients dropped out after inclusion. Three patients (4 eyes) who were unable to complete the designed follow-up, 4 patients (6 eyes) whose retroillumination images could not be analyzed due to the small size of their pupils or iris synechiae and 1 patient (1 eye) with a high intraocular pressure after surgery were excluded from the analysis. Additionally, 2 eyes in Group A, 1 eye in Group B and 1 eye in Group C received Nd: YAG laser capsulotomy due to serious VAO during the follow-up (Fig. 1). Therefore, 26 patients (30 eyes) were available for the final analysis. There were 7 eyes in Group A, 10 eyes in Group B and 13 eyes in Group C. The mean patient age was 6 ± 2.76 months (range, 3–16 months) and was comparable in the three groups. The follow-up time was 18 ± 2.49 months (range, 13–24 months) and was comparable in the three groups.

### Mean ACO and PCO Areas

**Figure 2:**
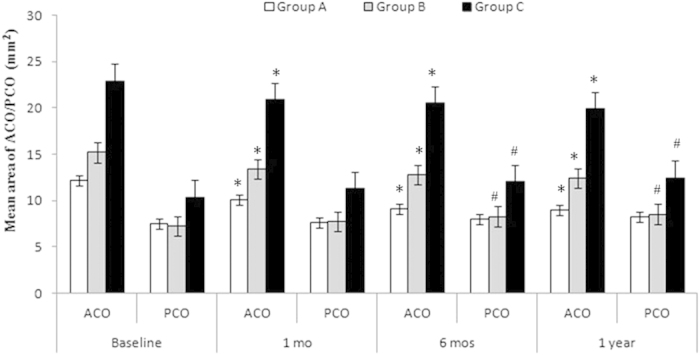
Mean area of the ACO and PCO at baseline and at the postoperative visits. The area of the ACO decreased and the area of the PCO increased postoperatively. There was a significant difference in the area of the ACO between the baseline and the postoperative visits (*p < 0.05). However, for the area of the PCO, the only significant differences occurred in Groups B and C between the baseline and at 6 and 12 months postoperatively (^#^p < 0.05). Notes: ACO = anterior capsular opening; PCO = posterior capsular opening; mo = month.*p < 0.05, ^#^p < 0.05.

The mean areas of the ACO in Group A were 12.16 ± 0.71, 10.09 ± 0.43 (p = 0.009 compared with baseline), 9.08 ± 0.53 (p = 0.005), and 9.00 ± 0.85 (p = 0.001) mm^2^ at baseline and postoperative 1 month, 6 months, and 1 year, respectively. The ACOs of Group B were 15.22 ± 1.18, 13.43 ± 0.97 (p = 0.007), 12.79 ± 1.00 (p = 0.003), and 12.41 ± 1.09 (p = 0.002) mm^2^, respectively; those of Group C were 22.92 ± 1.45, 20.86 ± 0.87 (p = 0.028), 20.49 ± 1.04 (p = 0.014), and 19.88 ± 0.81 (p = 0.020) mm^2^, respectively. For all three groups, the mean area of the ACO decreased significantly postoperatively (all p < 0.05, as above) (Fig. 2).

The mean areas of the PCO in Group A were 7.55 ± 0.49, 7.64 ± 0.78 (p = 0.790), 8.00 ± 0.66 (p = 0.185), and 8.22 ± 0.74 (p = 0.101) mm^2^ at baseline and postoperative 1 month, 6 months, and 1 year, respectively. The PCOs of Group B were 7.26 ± 1.21, 7.75 ± 1.35 (p = 0.078), 8.29 ± 1.21 (p = 0.045), and 8.53 ± 1.25 (p = 0.033) mm^2^, respectively, and those of Group C were 10.37 ± 2.91, 11.29 ± 2.49 (p = 0.086), 12.07 ± 2.86 (p = 0.001), and 12.46 ± 2.95 (p = 0.005) mm^2^, respectively. For all three groups, the mean area of the PCO increased postoperatively (Fig. 2).

### Area Change in ACO and PCO

**Figure 3:**
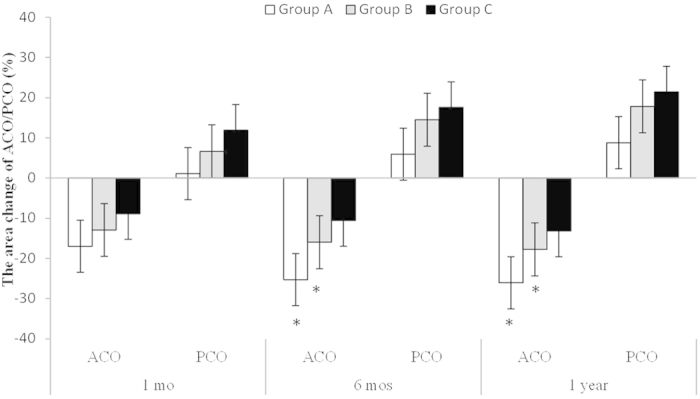
Changes in the areas of the ACO and PCO at 1 month, 6 months and 1 year after surgery. Notes: ACO = anterior capsular opening; PCO = posterior capsular opening; mo = month.*p < 0.05.

The percentage decreases of the ACO in Group A at 1 and 6 months and 1 year after surgery were 16.97 ± 1.93, 25.26 ± 2.19, and 26.04 ± 2.85%, respectively; in Group B, the respective values were 12.92 ± 1.73, 15.95 ± 0.60, and 17.74 ± 1.09%; and in Group C, the respective values were 8.88 ± 2.17, 10.55 ± 1.66, and 13.18 ± 2.60%. Group A had the highest capsule constriction, and there was a significant difference among the three groups at the various time points (p = 0.007, 0.000, and 0.001, respectively). The percentage reduction in the ACO area increased with time, and the differences between 6 months, 1 year and 1 month were significant in Group A (p = 0.008 and 0.010, respectively) and Group B (p = 0.045 and 0.015, respectively). The differences among the various visits in Group C were not significant (p = 0.351 and 0.093, respectively) (Fig. 3).

The percentage increases in the PCO in Group A at 1 and 6 months and 1 year after surgery were 1.12 ± 6.63, 5.93 ± 5.13, and 8.80 ± 4.98, respectively; the respective values in Group B were 6.67 ± 3.35, 14.52 ± 5.95, and 17.84 ± 6.24; and the respective values in Group C were 11.94 ± 5.44, 17.59 ± 6.26, and 21.45 ± 6.73. Group C had the highest posterior capsule enlargement compared with the other two groups, although the differences did not reach statistical significance (p = 0.119, 0.110, and 0.098, respectively). Furthermore, there was no significant difference among the various postoperative visits in any of the groups (Fig. 3).

Overall, Group A had the highest capsule constriction and percentage reduction, which increased with time. There was a significant difference in the percentage reduction between 6 months, 1 year and 1 month in Group A and Group B (*p < 0.05). Group C had the highest posterior capsule enlargement, although the difference did not reach statistical significance.

### Ratio of Opacity Accounting for PCO

**Figure 4:**
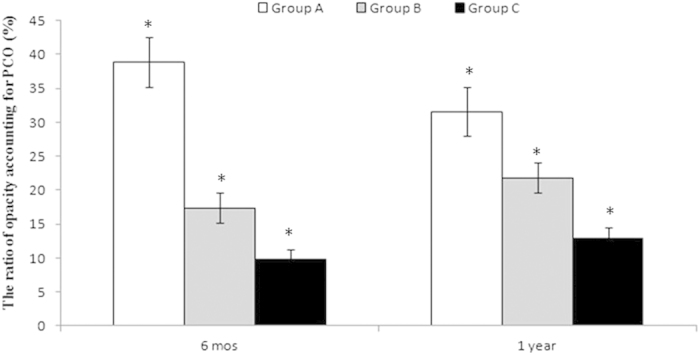
Proportion of opacity accounting for the PCO at 6 months and 1 year after surgery. Notes: PCO = posterior capsular opening; mo = month.*p < 0.05. The percentage of participants with PCOO was the highest in Group A, compared with the two other groups. There was a significant difference between 6 months and 1 year after surgery in all of the groups (*p < 0.05).

The percentage of PCOO was highest in Group A compared with Groups B and C (p = 0.001 and 0.001, respectively). There was a significant difference between 6 months and 1 year after surgery in Groups A, B and C (p = 0.005, 0.043 and 0.002, respectively; Fig. 4). In total, 2 eyes in Group A, 1 eye in Group B and 1 eye in Group C underwent Nd: YAG laser capsulotomy due to serious VAOs from 1 month to 16 months. The incidences of serious VAOs were 22.25% (2/9), 9.1% (1/11), and 7.1% (1/14) in Groups A, B and C, respectively. Group A had the highest rate of serious VAOs, although there was no significant difference among the three groups (p = 0.519).

## Discussion

This randomized controlled trial (RCT) investigated the capsular outcomes of pediatric cataract surgery performed with different anterior capsulorhexis sizes. We found that a diameter of 4.0–5.0 mm yielded optimal capsular outcomes, based on its relatively moderate contraction of ACO, moderate enlargement of PCO, and lower percentage of PCOO and VAO. To the best of our knowledge, this is the first RCT to investigate the relationship between the primary capsulorhexis size and capsular outcomes after pediatric cataract surgery.

Currently, primary anterior and posterior capsulectomy and anterior vitrectomy are the main surgical interventions for pediatric cataracts, particularly among young children^15^. The decision regarding whether to implant an IOL at the time of cataract surgery in infants, or leave the child aphakic with the secondary IOL implantation later in childhood, is controversial. However, the study of the Infant Aphakia Treatment Study (IATS) showed that for infants, cataract surgery without IOL implantation is an optimal choice, as it decreases the incidence of VAO and the need for second interventions, as well as decreasing costs^4^,^5^. Furthermore, ideal capsular outcomes are attained, and secondary in-the-bag IOL implantation can retain the normal anatomic position of the IOL while reducing uveal irritation and chronic inflammation^8^,^9^. For age-related cataracts, many studies have clarified that the optimal anterior capsulorhexis diameter is slightly smaller than the diameter of the simultaneously implanted IOL optic surface, with 0.5–1.0 mm capsulorhexis edges covering the IOL optic surface^16^. However, the optimal size of the anterior capsulorhexis that should be used to obtain superior capsular outcomes for secondary IOL implantation in primary pediatric cataract surgery has not yet been reported.

Several devices have been reported to control the diameter and shape of capsulorhexis, such as capsulotomy diameter marks, ring-shaped calipers, or the data-injection system^17^,^18^,^19^,^20^. Some of these devices require specific microscopes and a sophisticated dedicated instrument. In this study, we designed a needle with 1-mm scale laser markers as a calibration tool to verify the diameter of the capsulorhexis. Our needles are made from viscoelastic needles that have been processed with lasers and that can be autoclaved for repeated usage. During the surgery, we used the needle before and after the capsulorhexis to confirm the capsulorhexis size. Compared with the other devices, this self-made needle calibration system is inexpensive, convenient and effective. Moreover, an available method for objective quantification of capsular outcomes during follow-up is another important issue. Therefore, we also developed the imaging software ECO, a custom image-analysis algorithm using MATLAB (The MathWorks, Inc., Natick, MA, USA) to quantitatively measure capsular outcomes, which was determined to be user-friendly in our previous study and the current study. As is known that the pediatric capsule is highly elastic, making it difficult to perform capsulorhexis with a bent needle or forceps. Therefore we used radiofrequency diathermy to open the anterior and posterior capsule, obtaining a success rate greater than 95%. This is a simple, effective, and excellently safe procedure. Burkhard Dick *et al.*^21^ recently reported the safety of femtosecond laser-assisted capsulotomy in pediatric cataract surgery. However, it is not to date sufficiently inexpensive for clinical use in developing countries. Many factors can affect the constriction of the ACO after cataract surgery^22^. In our study, Group A (3.0–3.9 mm anterior capsulorhexis in diameter) had the most pronounced ACO constriction, and there were significant differences among the three groups at their postoperative visits. The constriction process of the ACO began postoperatively at 1 month and continued until 1 year. In general, Davison suggested that the capsule contraction was caused by an imbalance between the centrifugal and centripetal forces acting on the capsular bag^23^. However, the associations between the initial ACO size and the anterior capsule constriction remain unclear. Joo *et al.*^24^ reported that the initial ACO size might play a role in capsule contraction. However, Cochener *et al.*^25^ found insignificant relationships between ACO size and postoperative constriction, partly due to the small variation in their observed ACO sizes. These conclusions were drawn only from studies of age-related cataracts.

As opposed to the ACO, for the initial PCO, a 3.0-mm diameter capsulorhexis was well accepted and had a tendency to widen. Michael *et al.*^26^ demonstrated that the PCO was enlarged in a group undergoing silicone IOL implantation. We also noted the PCO enlargement after pediatric cataract surgery, even without IOL implantation in this pediatric study population. The PCO change in Group A was relatively minimal, whereas Group C had the highest posterior capsule enlargement, although the difference did not attain statistical significance. The reason for this finding may lie in the reduced PCO centrifugal force due to the serious constriction and fibrosis of the anterior capsule and the fusion of the anterior and posterior capsules, which reflect the dynamics of the capsular bag. The percentage of PCOO and the incidence of a second intervention due to VAO was highest in Group A compared with the other two groups. This finding may be due to the higher rate of residual lens epithelial cells (LECs) in the group with the small capsulorhexis opening. Group A had the highest rate of VAO, although no significant difference was found among the three groups due to the small number of patients enrolled in our study.

The results and interpretation of the current study must be understood within the context of its strengths and limitations. The strengths of the study include its randomized controlled design, the performance of the surgeries by a single experienced pediatric cataract surgeon using radiofrequency diathermy with a self-made needle calibration that ensured accuracy of the three controlled groups at baseline, and the investigation’s adherence to a unified and strict postoperative regimen and follow-up protocol within the same study group (CCPMOH). The weaknesses of the study must also be acknowledged. The sample size of the study groups was relatively small, and the results were most likely slightly biased by the exclusion of patients with serious iris synechiae and patients whose pupils could not dilate after surgery. This study only evaluated aphakic rather than pseudophakic eyes, and our findings could not be applied to pseudophakic eyes. However, the aim of this paper is to investigate the relationship between primary capsulorhexis size and capsular outcome of aphakic patients and eventually provide the theoretical basis to the successful in-the-bag IOL implantation at the second surgery. We employed a self-made calibrated needle in the chamber to measure the size of the capsule openings during surgery, but these measurements were made externally and postoperatively. Because of corneal magnification, immediate postoperative readings would be larger than intraoperative readings, yielding another potential bias. Despite these limitations, this study remains one of the first to investigate the relationship between the primary capsulorhexis size and capsular outcomes after pediatric cataract surgery.

There are important potential implications for our present findings. Our research has demonstrated that an ideal capsulorhexis size for pediatric cataracts not only would decrease the rates of capsular complications, including capsule contraction and VAO, but also would increase the probability of successful in-the-bag IOL implantation at the second surgery. We are currently following the pediatric patients from our study to assess their rates of accurate in-the-bag IOL implantation.

## Methods

### Patients

Forty-one congenital cataract patients <2 years old were recruited from Zhongshan Ophthalmic Center (ZOC) between March 2011 and November 2012 as registered members of the Childhood Cataract Program of the Chinese Ministry of Health (CCPMOH)^27^. The research protocol was approved by the Institutional Review Board/Ethics Committee of the Sun Yat-sen University. For more details, refer to Protocol S1 and Checklist S1 in the supplementary information. Informed written consent was obtained from at least one parent of each participating child, and the tenets of the Declaration of Helsinki were followed throughout this study. To make the Evaluation of Capsular Outcomes (ECO) software used in this study confidential, this study was registered with the Clinical Research Internal Management System of ZOC before participant enrollment began. Consequently, the study was registered after the enrollment of participants started, with Clinical-Trials.gov, (https://www.clinicaltrials.gov, registration number: NCT02158325, First received date: June 4, 2014). The authors confirm that all ongoing and related trials for this study are registered. Patients with glaucoma, ocular trauma, corneal disorders, persistent hyperplastic primary vitreous, rubella, Lowe syndrome, capsular fibrosis, or surgical complications, as well as those whose pupils could not dilate normally postoperation or who could not complete the follow-up, were excluded. Three patients were excluded, and thirty-eight patients (45 eyes) were included in this study (Fig. 1).

### Randomization Grouping

Preoperatively, all patients were randomly divided into 3 groups (Group A had 13 patients and 15 eyes, Group B had 13 patients and 15 eyes, and Group C had 12 patients and 15 eyes) using a random number chart and underwent cataract surgery without IOL implantation. The patients in Group A received a small anterior capsulorhexis that was 3.0 mm to 3.9 mm in diameter, Group B received a medium capsulorhexis that was 4.0 mm to 5.0 mm in diameter, and group C received a large capsulorhexis that was 5.1 to 6.0 mm in diameter.

### Surgical Technique

**Figure 5:**
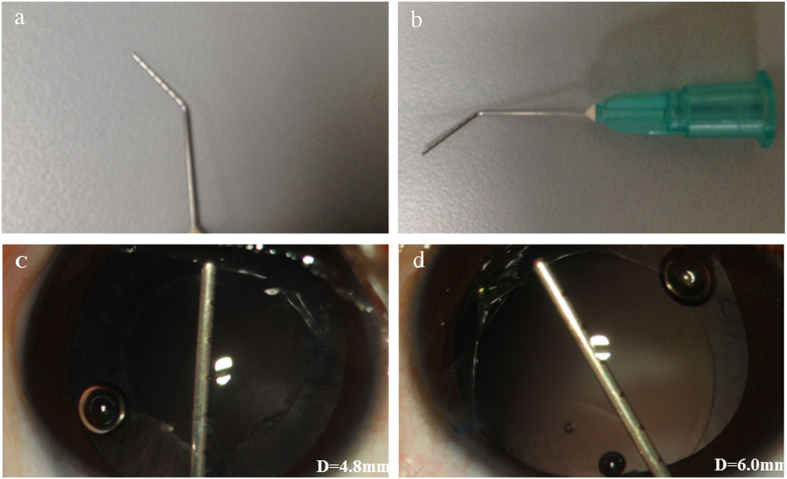
The self-made needle with laser marker (1-mm scale) used to measure and confirm the capsulorhexis diameter during surgery. (**a**) The eight laser markers can clearly be seen on the needle. (**b**) The entire needle with laser marker is shown. (**c**) A 5.0-mm capsulorhexis was completed and confirmed with the laser-marker needle. (**d**) A 6.0-mm capsulorhexis was completed and confirmed with the laser-marker needle. Notes: D = Diameter.

All patients underwent cataract surgery performed by a single experienced pediatric cataract surgeon (YZL) using a technique that has been previously described^28^ and is summarized briefly below. After a conjunctival peritomy was performed, a superior scleral tunnel incision was created using a 3.2-mm keratome. An anterior central curvilinear capsulotomy was made using radiofrequency diathermy. The anterior capsulorhexis size differed among the three groups. Before and after capsulorhexis, a self-made calibrated needle with a laser marker (1-mm scale) (Xinkeling, Inc., Shanghai, China) was used to measure and confirm the capsulorhexis diameters (Fig. 5). The nucleus and cortex were removed using the irrigation/aspiration device of the Infiniti Vision System (Alcon Laboratories, Fort Worth, TX, USA). A posterior central curvilinear capsulotomy incision that was 3.0-mm in diameter was made, also using radiofrequency diathermy. Then, a limited anterior vitrectomy was performed using the Infiniti Vision System vitrectomy instrument. The tunnel incision was sutured with 10–0 nylon sutures. All patients received subconjunctival dexamethasone (2 mg) during surgery, and all surgeries were performed under general anesthesia.

### Postoperative Regimen and Follow-up Protocol

Postoperatively, Tobradex eye drops (tobramycin 0.3%, dexamethasone 0.1%, Alcon) were used 6 times per day, and Tobradex eye ointment (tobramycin 0.3%, dexamethasone 0.1%, Alcon) was applied once per night for 2 weeks. From 2 weeks to 1 month postoperatively, the eye drops were used 4 times per day. For the second postoperative month, the patient switched to pranoprofen eye drops 4 times per day (Senju Pharmaceutical Co., Ltd., Osaka, JP). This follow-up protocol was also used in our previous study. All patients were examined at 1 week, 1 month, 3 months, 6 months, 1 year, 18 months, and two years after surgery. Patients were followed for two years or until the development of severe VAO requiring Nd: YAG laser capsulotomy, whichever came first. The examinations consisted of visual acuity, IOP measurement by a Tonopen tonometer (Reichert, Inc., Seefeld, Germany), fundoscopy, an assessment by high-resolution digital retroillumination imaging (detailed protocol presented below).

### Slit-Lamp-Adapted Anterior-Segmental Photography

All of the postoperative pediatric patients underwent pupil dilation with compound tropicamide eye drops (tropicamide 0.3%, phemylephrine hydrochloride 0.3%, Zhongshan Ophthalmic Center, Guangzhou, China) 3–5 times (once every 10 minutes) until the pupil size was larger than 6 mm. Then, the children underwent slit-lamp-adapted anterior-segmental photography (BX900, Haag-Streit AG, Köniz, Switzerland) for each operated eye, including one digital coaxial retroillumination photograph (DCRP), one diffuse light photo and one slit-light photo across the central visual axis. For uncooperative children, a set of assistance procedures for slit-lamp-adapted photography was developed to instruct the parents to help prepare the child’s head position and to help the child open his or her eyes as described in our previous studies^11^,^28^. In brief, we developed a transformable bed for assisting pediatric ophthalmic examination (US Patent No.US 9,015,882 B2). We performed examinations after administering a sedative drug (i.e., chloral hydrate as a sleep aid) or under general anesthesia (if chloral hydrate was contraindicated or unacceptable) for children who were uncooperative to allow for regular examinations in the clinic.

### Division and Definition of ACO, PCO, PCOO and VAO

**Figure 6:**
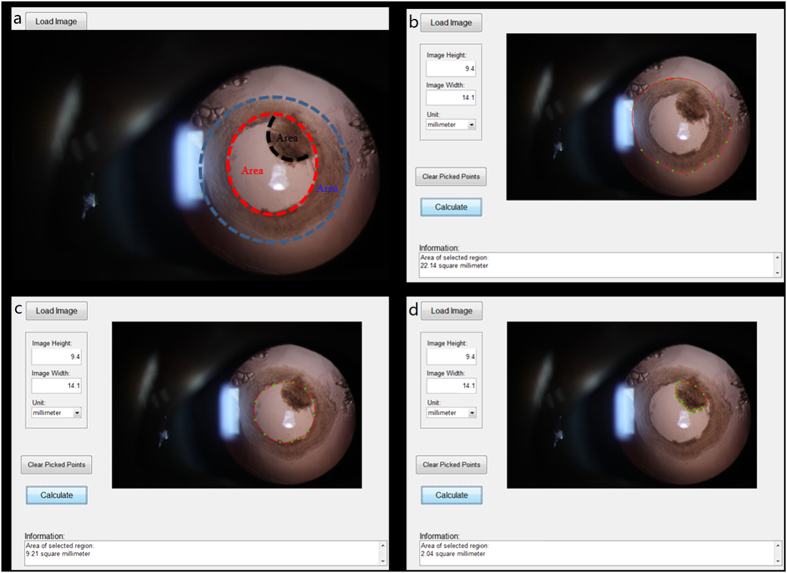
Division and definition of different capsule areas on standardized DCRPs. (**a**) Contour of different capsule areas in the retroillumination image, including the ACO (blue area), PCO (red area) and PCOO (black area). (**b**) The anterior capsule opening (ACO) area, (**c**) Posterior capsule opening (PCO) area, (**d**) Posterior capsule opening opacity (PCOO) area were traced by the ECO software. (Notes: DCRPs = digital coaxial retroillumination photographs; ACO = anterior capsular opening; PCO = posterior capsular opening; PCOO = posterior capsular opening opacification).

The postoperative capsular DCRPs were divided and defined as follows: area of the ACO, area of the PCO and area of the posterior capsule opening opacity (PCOO) (Fig. 6). The ACO and PCO are self-explanatory. PCOO indicates lens epithelium proliferation within the posterior capsule opening area causing prominent opacity. VAO is defined as opacity of the anterior and/or posterior capsule or lens epithelium proliferation within a 3-mm pupil size, which completely occludes light transmission across the pupil. If the fundus cannot be clearly viewed through the visual axis, the incidence of VAO is identified, and Nd: YAG laser is prescribed to eliminate the opacity of visual axis.

### Measurement of Outcomes

The primary outcomes were the areas of the ACO and PCO at different postoperative follow-up visits. The secondary outcomes were the percent reduction or increase in the ACO, PCO and PCOO at different postoperative follow-up visits and the total incidence of VAO. We also assessed surgical complications, such as capsular tears, inflammation, corneal edema, glaucoma, iris synechiae and postoperative macular edema. The surgery was video recorded, and pictures of the completed ACO and PCO were taken intraoperatively. Both the intraoperative and postoperative pictures were standardized and documented on an analysis computer using our ECO software, a custom image analysis algorithm developed by our team using MATLAB (The MathWorks, Inc., Natick, MA, USA). The contours of the ACO, PCO and PCOO were traced by hand using the ECO image processing software. Next, the capsule opening area, capsulorhexis diameter and opacity area were measured by computer. Two researchers independently used the software to quantify the values of the ACO, PCO and PCOO, and the results are the mean values determined by the two researchers. The mean values of the ACO, PCO and PCOO of each capsular type were calculated. The mean areas of the PCOO were analyzed at 6 months and 1 year. The percentage of PCOO was calculated as follows: PCOO area × 100/PCO area at the same time. If the patient underwent the Nd: YAG laser capsulotomy or a secondary surgical intervention due to serious VAO, the percentage of PCOO was recorded as 100%.

### Data Analysis and Statistics

The SPSS software package (version 17.0, SPSS, Inc., Chicago, IL, USA) was used for statistical analysis. The sample size calculation was based on power analysis. Power analysis adopts a hypothesis-testing method to determine the sample size according to several parameters, which include the pre-specified significance level, desired power level and expected effect size. Assuming a two-tailed alpha of 0.05, a probability of 0.2 for beta error (80% power) and an effect size of 0.28 after calculating with respect to the same primary outcome measure (area of the ACO) from the result of our preliminary research, 10 participants per group were required. A paired T test was used to compare the areas of the ACO and PCO between the postoperative visits and baseline. ANOVA was used for changes in the ACO and PCO areas among the three groups at different postoperative visits. Due to the non-normal distribution, Kruskal-Wallis nonparametric tests were used to compare the area percentage of PCOO among the three groups, and the Wilcoxon signed rank test was used to compare the difference between the postoperative visits. All statistical tests were two-tailed, with α = 0.05. A p value of <0.05 was considered statistically significant.

## Additional Information

**How to cite this article**: Lin, H. *et al.* Capsular Outcomes Differ with Capsulorhexis Sizes after Pediatric Cataract Surgery: A Randomized Controlled Trial. *Sci. Rep.* **5**, 16227; doi: 10.1038/srep16227 (2015).

## Supplementary Material

Supplementary Information 1
