# Supplementary material for: Capsular Outcomes Differ with Capsulorhexis Sizes after Pediatric Cataract Surgery: A Randomized Controlled Trial
Source: Sci Rep. 2015 Nov 5;5:16227. doi: 10.1038/srep16227 (PMC4633668; doi:10.1038/srep16227)
Supplement: Supplementary Information 1 [file srep16227-s1.pdf]

Capsular Outcomes Differ with Capsulorhexis Sizes after Pediatric Cataract Surgery: A Randomized Controlled Trial

Study Protocol

Table of Contents

1. Background and Purpose…………………………………………………………...2

2. Patient Recruitment and Enrollment ……………………………………………….5

3. Surgical Protocols ……………………...…………………………………………….7

4. Follow-up and evaluation of surgical outcomes……………………………………....9

5. Statistical Analyses…………………………………………………………………10

6. References………………………………………………………………………….11

Chapter 1

Background and Purpose

1.1 Objectives

This study is a randomized clinical study with the following objectives:

- To compare the capsular outcomes of pediatric cataract surgery performed with different anterior capsulorhexis sizes.
- To investigate the relationship between the primary capsulorhexis size and the capsular outcome.

1.2 Rationale of the Study

The study is conducted for the following reasons:

- In our previous study, we found that prior to performing a secondary in-the-bag IOL implantation, an ideal capsular outcome for the anterior/posterior capsulorhexis opening (ACO/PCO) should be achieved after the primary surgery.

- Capsular outcomes of anterior/posterior capsulorhexis opening (ACO/PCO) in aphakic eye after pediatric cataract surgery are essential in the success of performing a secondary in-the-bag intraocular lens implantation. However, the relationship between the primary capsulorhexis size and the capsular outcome is not well characterized.

- Investigation of this relationship may help improve success rate of secondary in-the-bag intraocular lens implantation and visual prognosis.

1.3 Summary of Study Protocol

Major eligibility criteria:

- Congenital cataract patient registered in the Childhood Cataract Program of the Chinese Ministry of Health (CCPMOH)
- < 2 years old
- Patients without glaucoma, ocular trauma, corneal disorders, persistent hyperplastic primary vitreous, rubella, Lowe syndrome, capsular fibrosis, or surgical complications
- Whose pupils could dilate normally postoperation
- Who could complete the follow-up were excluded.

Treatment groups: Eligible patients underwent cataract surgery performed by a single experienced pediatric cataract surgeon (YZL) using a technique that has been previously described and which is summarized briefly below. After a conjunctival peritomy was performed, a superior scleral tunnel incision was created using a 3.2-mm keratome. An anterior central curvilinear capsulotomy was made using radiofrequency diathermy. The anterior capsulorhexis size differed among the three groups. Before and after capsulorhexis, a self-made calibrated needle with a laser marker (1-mm scale) (Xinkeling Inc., Shanghai, China) was used to measure and confirm the diameters of the capsulorhexis (Figure 2). The nucleus and cortex were removed using the irrigation/aspiration device of the Infiniti Vision System (Alcon Laboratories, Fort Worth, TX, USA). A posterior central curvilinear capsulotomy incision that was 3.0-mm in diameter was made also using radiofrequency diathermy. Then, a limited anterior vitrectomy was performed using the vitrectomy

instrument of the Infiniti Vision System. The tunnel incision was sutured with 10-0 nylon sutures. All patients received subconjunctival dexamethasone (2 mg) during surgery, and all surgeries were performed under general anesthesia.

Examination Schedule: All patients were examined at 1 week, 1 month, 3 months, 6 months, 1 year, 18 months, and two years after surgery. Patients were followed for two years or until the development of severe VAO requiring ND: YAG laser capsulotomy, whichever came first. The examinations consisted of visual acuity, IOP measurement by a Tonopen tonometer (Reichert Inc., Seefeld, Germany), fundoscopy and an assessment by the Pentacam Scheimpflug Imaging System (Oculus, Germany). High-resolution digital retroillumination imaging of the capsule through dilated pupils (> 5 mm) was performed with the BX 900 anterior eye segment analysis system (Haag-Streit AG, Switzerland). Young children who were uncooperative during the ophthalmic examination received chloral hydrate (10%, 0.1 ml/Kg) as a sleep aid or received general anesthesia.

Primary Outcome: Mean areas of ACO, PCO, PCOO and their variation trends after surgery.

Secondary Outcomes: We assessed surgical complications, such as capsular tears, inflammation, corneal edema, glaucoma, iris synechia and postoperative macular edema.

Chapter 2

Patient Recruitment and Enrollment

2.1 Participant Recruitment

Registered members of the Childhood Cataract Program of the Chinese Ministry of Health (CCPMOH) were recruited from Zhongshan Ophthalmic Center (ZOC) between March 2011 and November 2012.

2.2 Exclusion Criteria

Patients will be excluded from the study if they meet any of the following criteria:

- >= 2 years old
- History of glaucoma, ocular trauma, corneal disorders, persistent hyperplastic primary vitreous, rubella, Lowe syndrome, capsular fibrosis, or surgical complications
- Whose pupils could not dilate normally postoperation
- Who could not complete the follow-up were excluded.

2.3 Examination Procedures

Ophthalmic examination will assess:

1. Visual acuity measured by Teller Visual Card
2. Pupil dilation and slit-lamp examination/photography
3. Pentacam scan
4. Non-contact specular microscopy
5. Non-contact or Tonopen tonometer

Surgical complications will assess:

1. Capsular tears
2. Inflammation
3. Corneal edema
4. Glaucoma
5. Iris synechia
6. Postoperative macular edema.

Chapter 3

Surgical Protocols

3.1 Treatment Groups

Patients will be assigned to one of three treatments:

1. Group A received a small anterior capsulorhexis that was 3.0 mm to 3.9 mm in diameter;
2. Group B received a medium capsulorhexis that was 4.0 mm to 5.0 mm in diameter;
3. Group C received a large capsulorhexis that was 5.1 to 6.0 mm in diameter.

3.2 Surgical Protocols

Surgery will be performed by a single surgeon (the PI, Dr. Liu).

After a conjunctival peritomy was performed, a superior scleral tunnel incision was created using a 3.2-mm keratome. An anterior central curvilinear capsulotomy was made using radiofrequency diathermy. The anterior capsulorhexis size differed among the three groups. Before and after capsulorhexis, a self-made calibrated needle with a laser marker (1-mm scale) (Xinkeling Inc., Shanghai, China) was used to measure and confirm the diameters of the capsulorhexis (Figure 2). The nucleus and cortex were removed using the irrigation/aspiration device of the Infiniti Vision System (Alcon Laboratories, Fort Worth, TX, USA). A posterior central curvilinear capsulotomy incision that was 3.0-mm in diameter was made also using radiofrequency diathermy. Then, a limited anterior vitrectomy was performed using the vitrectomy instrument of the Infiniti Vision System. The tunnel incision was sutured with 10-0 nylon sutures. All patients received subconjunctival dexamethasone (2 mg) during surgery, and all surgeries were performed under general anesthesia.

3.3 Postoperative Medical Therapy

Postoperatively, tobradex eye drops (tobramycin 0.3%, dexamethasone 0.1%, Alcon) were used 6 times per day, and tobradex eye ointment (tobramycin 0.3%, dexamethasone 0.1%, Alcon) was applied once per night for 2 weeks. From 2 weeks to 1 month postoperatively, eye drops were used 4 times per day. For the second postoperative month, the patient switched to pranoprofen eye drops 4 times per day (Senju Pharmaceutical Co., Ltd., Osaka, JP). This follow-up protocol was also used in our previous study.

Chapter 4

Follow-up and evaluation of surgical outcomes

4.1 Follow-up Examination Schedule

All patients were examined at 1 week, 1 month, 3 months, 6 months, 1 year, 18 months, and two years after surgery.

4.2 Follow-up Examination Procedures

Patients were followed for two years or until the development of severe VAO requiring ND: YAG laser capsulotomy, whichever came first. The examinations consisted of visual acuity, IOP measurement by a Tonopen tonometer (Reichert Inc., Seefeld, Germany), fundoscopy and an assessment by the Pentacam Scheimpflug Imaging System (Oculus, Germany). High-resolution digital retroillumination imaging of the capsule through dilated pupils (> 5 mm) was performed with the BX 900 anterior eye segment analysis system (Haag-Streit AG, Switzerland). Young children who were uncooperative during the ophthalmic examination received chloral hydrate (10%, 0.1 ml/Kg) as a sleep aid or received general anesthesia.

Chapter 5

Statistical Analyses

The SPSS software package (version 17.0, SPSS Inc., Chicago, IL, USA) was used for statistical analysis. A power analysis with a two-sided 5% level and a power of 80% were used for sample size calculation, and a sample size of 10 were necessary in each group. A paired-T test was used to compare the areas of the ACO and PCO between the postoperative visits and baseline. An ANOVA was used for the area changes in the ACO and PCO among three groups at different postoperative visits, and Bonferroni tests were used to compare the differences between any two groups. We also applied a generalized estimating equation with a working covariance matrix of unstructured correlations to analyze repeated measures data and the correlation effect of patients with both eyes. Kruskal Wallis nonparametric tests were used to compare the area percentage of PCOO among the three groups, and the Wilcoxon rank test was used to compare the difference between the postoperative visits. The percentage of VAO was compared using Fisher's exact test. All statistical tests were two-tailed with $\alpha = 0.05$. A p value of < 0.05 was considered statistically significant.

Chapter 6

References

1. Lin H, Chen W, Luo L, et al. Ocular hypertension after pediatric cataract surgery: baseline characteristics and first-year report. PLoS One. 2013;8(7):e69867.
2. Luo L, Lin H, Chen W, et al. In-the-bag intraocular lens placement via secondary capsulorhexis with radiofrequency diathermy in pediatric aphakic eyes. PLoS One. 2013;8(4):e62381.
3. Yan PS, Lin HT , Wang QL, Zhang ZP. Anterior Segment Variations with Age and Accommodation Demonstrated by Slit-lamp-adapted Optical Coherence Tomography. Ophthalmology. 2010. 117(12):2301-7.
4. Lin H, Chen W, Luo L, et al. Effectiveness of a short message reminder in increasing compliance with pediatric cataract treatment: a randomized trial. Ophthalmology. Dec 2012;119(12):2463-2470.
5. Lin H, Tan X, Luo L, Zhang X, Lin Z, Chen J, Wu H, Chen W, Zhong X, Qu B, Tang X, Zheng D, Chen W, Liu Y. Capsular outcomes after pediatric cataract surgery: qualitative classification and quantitative measurement. PloS one. 2014.
6. Lin H, Chen W, Luo L, et al. Ocular hypertension after pediatric cataract surgery: baseline characteristics and first-year report. PLoS One. 2013;8(7):e69867.
